# Perceptions about dementia clinical trials among underrepresented populations: a nationally representative survey of U.S. dementia caregivers

**DOI:** 10.1186/s13195-024-01579-5

**Published:** 2024-10-15

**Authors:** Brandon Leggins, Danielle M. Hart, Ashley J. Jackson, Robert W. Levenson, Charles C. Windon, Jennifer Merrilees, Winston Chiong

**Affiliations:** 1 Department of Neurology, University of California San Francisco, San Francisco, CA USA; 2 Department of Psychology, University of California Berkeley, Berkeley, CA USA

**Keywords:** Clinical trials, Dementia, Caregiving, National survey, Recruitment, Alzheimer’s disease

## Abstract

**Background:**

The research community has historically failed to enroll diverse groups of participants in dementia clinical trials. A unique aspect of dementia care research is the requirement of a study partner, who can attest to the care recipient’s clinical and functional capacity. The aim of this study is to assess racial and ethnic differences and the importance of various trial considerations among dementia caregivers, in their decision to participate in clinical research as study partners.

**Method:**

We embedded a vignette about a hypothetical dementia clinical trial in a nationally representative survey of U.S. dementia caregivers, oversampling non-Hispanic Black and Hispanic caregivers. Dementia caregivers were asked about their willingness to participate in the trial with their care recipient and rated the importance of nine considerations in hypothetical decisions to participate. Caregiver demographic characteristics were analyzed as predictors of trial participation in a base demographic model. In a second reasons model caregiver demographic characteristics and the rated importance of the nine considerations were separately analyzed as predictors; both models used survey-weighted logistic regression.

**Result:**

The sample consisted of 610 dementia caregivers, including 156 non-Hispanic Black and 122 Hispanic caregiver participants. In the base demographic model, hypothetical trial participation was negatively associated with older caregiver age (OR (odds ratio) = 0.72, p = < 0.001). In the reasons model, the rated importance of a social responsibility to help others by participating in research was significantly associated with participation (OR = 1.56, *p* = 0.049), while the importance of the possibility of the care recipient experiencing serious side effects was negatively associated with participation (OR = 0.51, *p* = 0.003). In both models there was no significant difference in hypothetical participation between non-Hispanic Black and non-Hispanic White caregivers, or between Hispanic and non-Hispanic White caregivers.

**Conclusion:**

Hispanic and non-Hispanic Black dementia caregivers were not less likely than non-Hispanic White dementia caregivers to participate in a hypothetical dementia clinical trial. Our study suggests that failures to recruit diverse populations in dementia clinical research are not attributable to less willingness among members of underrepresented groups but may instead reflect structural barriers and historic exclusion from trial participation.

**Supplementary Information:**

The online version contains supplementary material available at 10.1186/s13195-024-01579-5.

## Introduction

Newly approved anti-amyloid treatments to slow the progression of Alzheimer’s disease have been heralded as a “step forward” towards a future era of more effective disease modifying therapies for dementia [1]. However, the research community has historically failed to enroll a diverse groups of participants in dementia clinical trials [2, 3], raising concern that populations most affected by dementia may be denied the benefits of society’s investment in dementia clinical research. As examples, Hispanic Americans and non-Hispanic Black Americans are nearly twice as likely to experience dementia compared to non-Hispanic White Americans [4, 5]; yet are underrepresented in dementia clinical trials. For example, the intervention arm of the pivotal trial of donanemab included only 19 Black participants (2.2%) and 35 Hispanic participants (5.7%) [6], samples too small to license inferences about relative efficacy or even safety. As structural and medical factors underlying increased risk for dementia in these groups (including undertreated chronic medical conditions, environmental injustice, experiences of racism including within the health care system, and other social determinants of health) could also influence the safety and efficacy of new treatments, dementia clinical trials that fail to improve their recruitment strategies and represent the U.S. population at risk for dementia may lack generalizability. Current recruitment strategies that target underrepresented populations have yet to be effectively evaluated [7]; on the other hand, it has been suggested that distrust of health professionals is a significant barrier in recruiting diverse populations, though evidence for this has been mixed [7–10].

A special feature of dementia clinical trials that influences enrollment is the requirement of a study partner, who accompanies the patient to visits and attests to the patient’s clinical and functional status [11, 12]. Family and friend caregivers’ willingness to serve as study partners, and their opinions about patients’ participation, are major factors in whether patients can feasibly participate. Therefore, in efforts to improve diversity in dementia clinical trials, researchers must consider the perspectives of family/friend dementia caregivers, who are needed as study partners and who often also assist in communicating and interpreting the patient’s own values in care and research [13].

Prior research has studied the impact of the type of study partner on dementia clinical trial enrollment, finding that spousal caregivers are disproportionally enrolled compared to non-spousal caregivers [14, 15]. Other work has documented dementia study partners’ perspectives on the burdens of research participation and considerations influencing willingness to enroll in clinical trials. A limitation of many of these studies is that they sample dementia caregivers who are already enrolled as study partners in Alzheimer’s disease research centers, and probably do not reflect the concerns of dementia caregivers who decline participation or, in many more cases, have never been approached for participation. Efforts to expand clinical research participation will depend crucially on the perspectives of caregivers who are not presently engaged in research.

In this preregistered study we embedded a vignette about a hypothetical dementia clinical trial in a nationally representative U.S. survey of dementia caregivers. To examine the concerns and perspectives of underrepresented dementia caregivers, we assessed for racial, ethnic, and other demographic differences in decisions about trial participation and estimated the influence of various clinical trial-related considerations (such as social responsibilities to participate in research, the risk of side effects, or the inconveniences of participation) on decisions to participate.

## Methods

### Design and subjects

As described in our preregistration (https://osf.io/uk3g7), this study was designed as a nationally representative cross-sectional survey of U.S. dementia caregivers. To enhance our ability to assess potential racial and ethnic differences in perspectives on dementia clinical trials, we deliberately oversampled Hispanic caregivers and non-Hispanic Black caregivers.

Participants for this study were recruited from the NORC AmeriSpeak panel. Operating at the University of Chicago, NORC AmeriSpeak is a large probability-based panel that is representative of the U.S. household population and has been widely utilized for public opinion polling and academic research. The NORC AmeriSpeak panel is built and maintained by applying area probability and address-based sampling based on the U.S. Census. A two-stage recruitment process is utilized to minimize nonresponse; households are recruited by mail and phone, and households that do not respond initially receive a follow-up including enhanced incentives and in-person field interviews. The panel provides sample coverage of at least 97% of the U.S. household population. Written informed consent was obtained from participants during enrollment in the AmeriSpeak Panel, and the NORC Institutional Review Board approved all panel recruitment and survey procedures.

To identify dementia caregivers in the NORC AmeriSpeak panel, we presented panel members with a dementia-specific screening question based on a stem used in five AARP/NAC Caregiving in the U.S. surveys from 1997 to 2020 [16]: “At any time in the last 12 months, have you provided unpaid care to a relative or friend 50 years or older who has Alzheimer’s disease, dementia, or other mental confusion, to help them take care of themselves? This may include helping with personal needs, household chores, money management, arranging services, or regular visits to see how they are doing. This person need not live with you.”

The study survey was fielded from July 22–August 8, 2022, by both the internet (including the mobile web) and telephone and was available in both English and Spanish.

### Caregiver measures

Standard demographic data on caregiver participants were provided by NORC from previous surveys and included caregiver gender, age, education, race/ethnicity, household income, rural/urban residence, and employment status. Caregiver gender was obtained by a self-report question with response options “female”, “male”, “transgender”, and “do not identify as male, female, or transgender.” In this manuscript we follow a contemporary distinction between sex and gender, instead using the categories “women” and “men” for gender.

Race and ethnicity was provided as a combined variable including 6 categories: White, non-Hispanic; Black, non-Hispanic; Other, non-Hispanic; Hispanic; 2+, non-Hispanic; and Asian, non-Hispanic. Participants who self-identified as non-Hispanic and as Native Hawaiian or Pacific Islander were included within the Asian non-Hispanic category. Participants who self-identified as non-Hispanic and as American Indian, Alaska Native, or another race not listed were included in the Other, non-Hispanic category.

Caregiver educational attainment was recorded in five categorical responses: less than high school, high school graduate, some college (vocational, tech school, associates), bachelor’s degree, and postgraduate study or professional degree. Employment status was reported in categorical responses: working as a paid employee, self-employed, temporary layoff, looking for work, retired, disabled, and other. Household income was reported in 16 categorical responses (such as $35,000 to $39,999).

NORC determines rurality by utilizing census tract and zip codes and calculating Rural-Urban Community Area (RUCA) codes. Participants with RUCA codes 4–10 were classified as rural, participants with RUCA codes 1–3 and who did not reside in the largest city in the area were classified as suburban, and participants with RUCA codes 1–3 and who resided in the largest city in the area were classified as urban.

### Care recipient measures

Characteristics of the people with dementia who were cared for by panel participants were not included in NORC’s preexisting data. We asked participants directly about their care recipients’ demographic and clinical characteristics such as age, gender, rurality, race/ethnicity, dementia stage, and dementia diagnosis.

Options for dementia diagnosis included Alzheimer’s disease, vascular dementia, Lewy body dementia, frontotemporal dementia, Parkinson’s disease, primary progressive aphasia, Huntington’s disease, dementia only (no further label), mild cognitive impairment, has not received a medical diagnosis, and has received a diagnosis unknown to the respondent. More than one option could be selected as many patients have received multiple diagnoses.

Dementia stage was categorized as very mild, mild, moderate, or advanced. These were determined using the Quick Dementia Rating System (QDRS)-derived Clinical Dementia Rating (CDR) [17]. Participants answered portions of the QDRS regarding their care recipient’s memory and recall, orientation, decision making and problem-solving abilities, activities outside the home, function at home and hobby activities, and toileting and personal hygiene, to which the CDR scoring rules were then applied [18].

### Relational measures

Relational characteristics were obtained by asking caregiver participants about their care recipient’s relationship to them, where the care recipient lived, whether they were the primary caregiver for the care recipient, and how frequently they visited the care recipient. To avoid collinearity with care recipient gender, care recipient relationship options were gender neutral. The options included the following: parent; spouse/partner; grandparent; sibling; another relative; and a friend, neighbor, or someone else who is not in the family. Caregivers described their care recipient’s residence as: in the caregiver’s home, a home with someone else, an independent living or retirement community, an assisted living facility where some care may be provided, a nursing care or long-term care facility, or somewhere else. Caregiver visit frequency options included more than once a week, once a week, a few times a month, once a month, few times a year, and less often. Primary caregiver status was designated to caregivers who either provided all the unpaid care, provided most of the unpaid care with help from one or more people, or split most of the care evenly with one or more people.

### Clinical trial vignette measurements

We assessed willingness to participate in a dementia clinical trial with a vignette describing a hypothetical clinical trial of a disease-modifying agent for dementia, with overall trial features and risks modeled after recent trials of anti-amyloid monoclonal antibodies (see Supplementary Sect. 1, Additional File 1). After reading the vignette, caregiver participants were asked if they would participate (or would have participated, for caregivers who had provided care in the preceding 12 months but were not currently providing care at the time of the survey) in this study with their care recipient with dementia. Responses were collected on a 4-point Likert scale (e.g., definitely yes, probably yes, probably no, definitely no). Respondents then rated the importance of nine considerations that could be reasons for either participating or not participating in the trial, informed by common themes of literature discussing incentives and barriers for dementia research participation [9, 14, 19, 20]: the possibility of direct clinical benefit, social responsibilities to participate in research, the possibility of benefiting one’s community, access to better caregiver support by being connected to experts, the risk of side effects, distrust of the drug company, the chance of receiving a placebo, concerns about the privacy of health information in a study, and trial-related inconveniences of travel and time commitments. Responses were collected on a 4-point Likert scale (e.g., not at all important, not very important, somewhat important, very important). The specific wording used is included in Table 1.

**Table 1:** Nine considerations that caregivers rated the importance of in their reasons for either participating or not participating in the hypothetical dementia clinical trial. Responses were collected on a 4-point likert scale (e.g., not at all important, not very important, somewhat important, very important)

Reasons for either participating or not participating in the clinical trial	
Clinical benefit for the care recipient	“There is a chance that your care recipient will get the drug and it could help them”
Social responsibility to participate in research	“We all have some responsibility to help others by volunteering for medical research”
Community benefit	“Participating in this study could help people in your specific community, by showing whether the drug works for people like your care recipient”
Caregiver support	“Participating in this study could help you take better care of your care recipient, by being connected to experts who can answer your questions”
Distrust of the drug company	“You wouldn’t trust the company that makes this drug”
Inconvenience	“It would be inconvenient to bring your care recipient to the research center each month”
Placebo	“There is a 50% chance your care recipient will get the placebo”
Side effects	“There is a chance your care recipient will have a serious side effect from the drug and will get hurt”
Privacy	“You are not sure that your information and your care recipient’s information will be kept private and confidential”

### Analysis

All analyses were conducted in R (version 4.4.0: 2024-04-24) using the RStudio environment and the *tidyverse*, *survey*, *srvyr*, *gtsummary*, *cowplot*, and *mediation* packages. Survey-weighted analyses were conducted using weights provided by NORC AmeriSpeak. As described in our preregistered analysis (https://osf.io/uk3g7), to assess for racial, ethnic, and other demographic differences in hypothetical decisions about clinical trial participation, we generated four nested survey-weighted logistic regression demographic models, with clinical trial participation (dichotomized to yes/no) as the outcome. To compare racial/ethnic differences, non-Hispanic White was used as the reference category because of our aim to assess potential differences between groups that are adequately represented and groups that are underrepresented. For similar reasons, urban and suburban respondents were grouped together for comparison with rural respondents. Prior to modeling and the dichotomization of the clinical trial participation outcome variable, Likert-scaled responses were examined across demographic groups to check for differences in the intensity of response (definitely/probably) that would not be captured by our logistic regression procedures.

Given the large number of potential predictors available for our analysis, we prespecified a model selection procedure to identify a model to parsimoniously fit our data in light of our primary study question about potential differences between underrepresented and represented populations in dementia clinical research. We specified four nested demographic models. The base demographic model (Model 1) included basic caregiver demographic characteristics as predictors: age (mean-centered and scaled by decade), gender, race/ethnicity (limited to Hispanic, non-Hispanic Black, and non-Hispanic White to avoid estimation errors due to small cell counts), and rurality. Model 2 included all predictors from the base model and added care recipient clinical characteristics: age, gender, race/ethnicity, rurality, dementia stage, dementia diagnosis (limited to Alzheimer’s disease, dementia only, MCI, no diagnosis, and diagnosis unknown to respondent to avoid estimation errors due to small cell counts, all entered independently as dichotomous variables), and whether dementia was the primary condition for which the care recipient required care. Model 3 included all predictors from Model 2 and added further caregiver characteristics: education, household income, and current employment status (simplified to working, retired, disabled and other unemployed). Model 4 included all predictors from Model 3 and added relational characteristics: care recipient relationship to caregiver (simplified to parent, spouse, other relative and non-relative to avoid estimation errors due to small cell counts), care recipient residence (simplified to in the same home as the caregiver, in a separate home, or in a facility), whether the caregiver was the sole/primary caregiver, and how frequently the caregiver visited the care recipient. Predictors in all four nested demographic models were tested for multicollinearity with a threshold GVIF of 5. Given our four nested demographic models, we then applied a prespecified model selection procedure to test whether the increased complexity of successive models was justified by improvements in model fit. Beginning with the base demographic model (Model 1), we sequentially assessed improvements in model fit from one model to the next more complex model using an ANOVA test and stopped if a significance threshold of *p* = 0.05 was not met.

To estimate the importance of various clinical trial-related considerations among dementia caregivers we generated another survey weighted logistic regression model, which we refer to as the reasons model. Caregivers’ ratings of the importance of considerations that could be reasons for participating (the possibility of direct clinical benefit, social responsibilities to participate in research, specific benefits to one’s own community, and caregiver support from researchers) or not participating (lack of trust, the possibility of being randomized to placebo, breach of confidentiality, the possibility of side effects, inconvenience) were inputted in the reasons model as predictors of clinical trial participation alongside base caregiver demographic characteristics (Model 1). For demographic predictors from Model 1 that were significantly associated with trial participation, we tested whether this effect was mediated by any of the trial consideration predictors (reasons model) in a causal mediation model.

### Exploratory tests of moderation

In addition to our preregistered analyses, in exploratory analyses we evaluated whether the influence of any clinical trial-related considerations on trial participation was moderated by race and ethnicity. As an initial test of moderation we modeled the interactions between such predictors and race/ethnicity; if significant at a threshold of *p* = 0.05 we assessed for potential differential effects of these predictors in subgroups restricted to Hispanic caregivers, non-Hispanic Black caregivers, and non-Hispanic White caregivers.

## Results

### Participant demographic characteristics

A total of 611 panel members who received the screening question indicated that they have provided unpaid care to someone who has Alzheimer’s disease, dementia, or other mental confusion, and all but one completed the embedded vignette (*n* = 610). As planned, Hispanic (*n* = 122) and non-Hispanic Black (*n* = 156) caregiver participants were oversampled. The demographic characteristics of the caregiver participants who completed the vignette are listed in Table 2.

**Table 2:** Demographic characteristics of dementia caregivers in the study sample

Characteristic	Hispanic, *N* = 122^*1*^	White, non-Hispanic, *N* = 297^*1*^	Black, non-Hispanic, *N* = 156^*1*^	2+, non-Hispanic, *N* = 18^*1*^	Asian, non-Hispanic, *N* = 9^*1*^	Other, non-Hispanic, *N* = 8^*1*^
**Caregiver rurality**						
Urban	58 (48%)	88 (30%)	93 (60%)	4 (22%)	4 (44%)	2 (25%)
Suburban	55 (45%)	140 (47%)	53 (34%)	10 (56%)	5 (56%)	4 (50%)
Rural	9 (7.4%)	69 (23%)	10 (6.4%)	4 (22%)	0 (0%)	2 (25%)
**Caregiver age (Mean ± sd)**	41 ± 15	52 ± 17	45 ± 17	42 ± 18	34 ± 7	40 ± 20
**Caregiver gender**						
Men	58 (48%)	120 (40%)	62 (40%)	6 (33%)	6 (67%)	5 (63%)
Women	64 (52%)	177 (60%)	94 (60%)	12 (67%)	3 (33%)	3 (38%)
**Caregiver education**						
Less than HS	11 (9.0%)	9 (3.0%)	16 (10%)	3 (17%)	1 (11%)	1 (13%)
HS graduate or equivalent	31 (25%)	53 (18%)	40 (26%)	3 (17%)	0 (0%)	4 (50%)
Vocational/tech school/some college/ associates	66 (54%)	130 (44%)	63 (40%)	10 (56%)	2 (22%)	2 (25%)
Bachelor’s degree	12 (9.8%)	55 (19%)	19 (12%)	2 (11%)	2 (22%)	0 (0%)
Post grad study/professional degree	2 (1.6%)	50 (17%)	18 (12%)	0 (0%)	4 (44%)	1 (13%)
**Relationship (who is the person you cared for)**						
Grandparent	31 (25%)	48 (16%)	28 (18%)	4 (22%)	4 (44%)	3 (38%)
Parent	34 (28%)	111 (37%)	51 (33%)	5 (28%)	2 (22%)	2 (25%)
Sibling	5 (4.1%)	10 (3.4%)	14 (9.0%)	0 (0%)	0 (0%)	0 (0%)
Spouse/partner	7 (5.7%)	23 (7.7%)	6 (3.8%)	0 (0%)	1 (11%)	1 (13%)
Another relative	18 (15%)	42 (14%)	24 (15%)	4 (22%)	2 (22%)	0 (0%)
A friend, neighbor, or someone else who is not in your family	24 (20%)	63 (21%)	30 (19%)	5 (28%)	0 (0%)	2 (25%)
**Primary Caregiver**	90 (74%)	208 (71%)	129 (84%)	10 (56%)	8 (89%)	8 (100%)

^*1*^ n (%); Mean ± SD

### Full likert-scaled responses

**Fig. 1:**
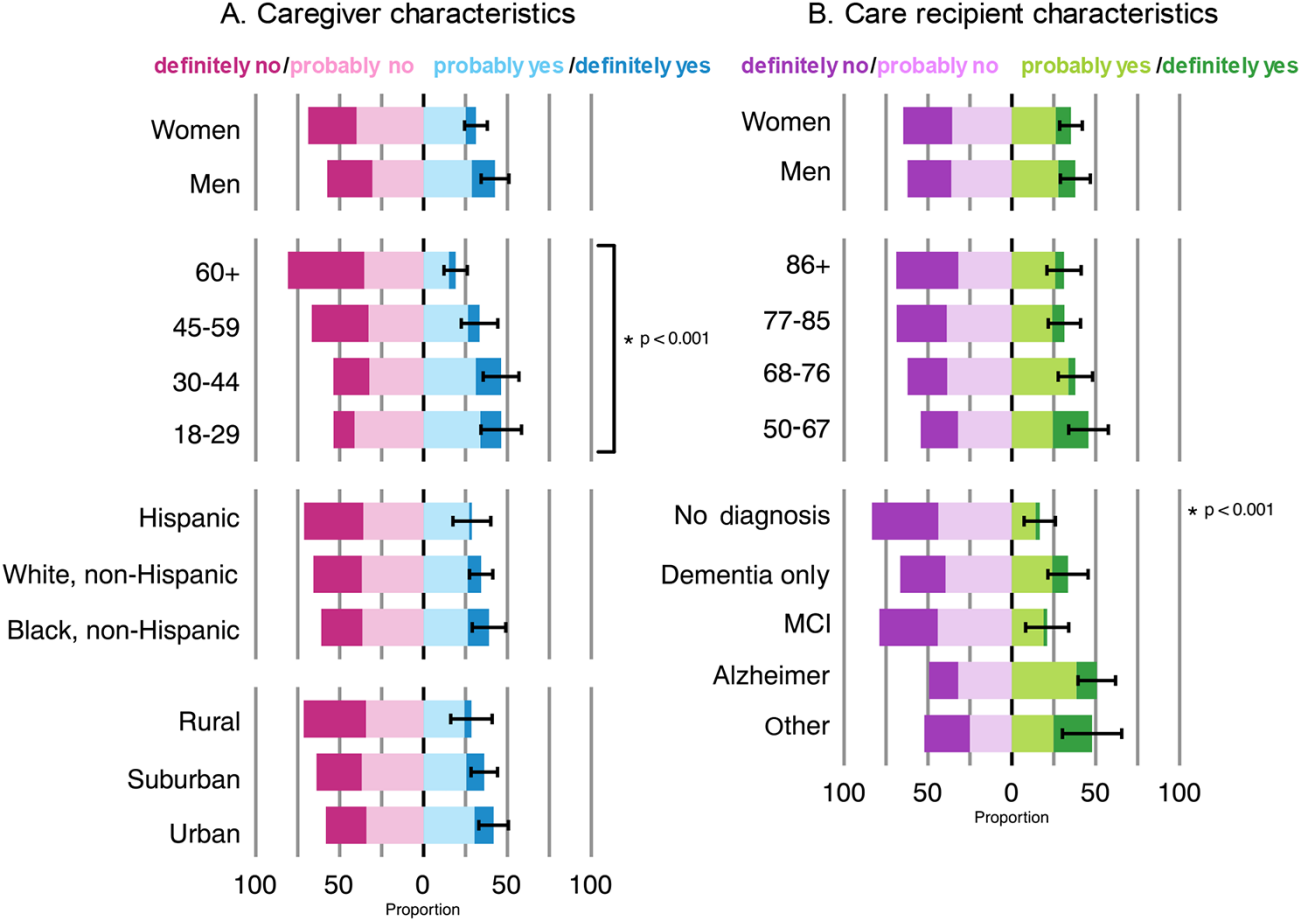
Survey weighted proportions of U.S. dementia caregivers’ willingness to participate in a hypothetical dementia clinical trial with their care recipient. Dementia caregivers responded to the following question: “Would you participate in this study with your care recipient?” Response options (from left to right) consisted of definitely no, probably no, probably yes, and definitely yes. **A**. Responses according to the characteristics of the dementia caregiver. **B**. Responses according to characteristics of the care recipient

Full Likert-scaled responses were examined across demographic groupings (Fig. 1). Across groups, 37% of all caregivers would participate in the described trial; 29% of Hispanic caregivers, 39% of non-Hispanic Black caregivers, and 34% of non-Hispanic White caregivers would participate. Younger caregivers were more willing to participate than older caregivers (Fig. 1); no such differences were apparent across groups defined by race/ethnicity or rurality.

### Model results

After collinearity testing, care recipient race/ethnicity was removed from Model 2 due to a GVIF value greater than our preregistered threshold of 5 (caregiver race/ethnicity remained in all models). Sequential ANOVA testing was performed on the four nested demographic models, and Model 2 was determined to be the preferred model among the nested models. From here on we refer to Model 2 as the adjusted demographic model.

### Base demographic model

In the base demographic model (Model 1), older dementia caregivers were less likely to participate in the clinical trial (by decade: OR (odds ratio) = 0.72, p = < 0.001). Non-Hispanic Black and Hispanic dementia caregivers were not less likely than non-Hispanic White dementia caregivers to participate in the clinical trial (Table 3); though the contrast between Hispanic and non-Hispanic White dementia caregivers approached statistical significance (OR = 0.49, *p* = 0.054). There were no significant differences observed for caregiver gender or rurality in the base model (Table 3).

Because the difference between Hispanic and non-Hispanic White caregivers was nearly significant in the base model while only small differences were apparent when inspecting responses across groups (Figs. 1 and 29% participation among Hispanic caregivers and 34% among non-Hispanic White caregivers), a single predictor model was examined in which the contrast between Hispanic and non-Hispanic White caregivers (OR = 0.77, *p* = 0.42), and the contrast between non-Hispanic Black and non-Hispanic White caregivers (OR = 1.22, *p* = 0.45) were not significant. Hispanic caregivers were over a decade younger on average than non-Hispanic White caregivers (41 years vs. 52 years, p = < 0.001); the difference in estimates for a model excluding age and a model including age indicates a suppression effect. (That is, Hispanic caregivers were not less likely to participate than non-Hispanic White caregivers overall, but when considering caregiver age, Hispanic caregivers were almost significantly less likely to participate than non-Hispanic White caregivers of similar age.) In an exploratory model testing interactions between age and race/ethnicity, the interaction between age and the contrast between Hispanic and non-Hispanic White caregivers was nonsignificant (OR = 1.15, *p* = 0.51), as was the interaction between age and the contrast between non-Hispanic Black and non-Hispanic White caregivers (OR = 0.90, *p* = 0.55).

### Adjusted demographic model

In the adjusted demographic model, older dementia caregivers were less likely to participate in the clinical trial (OR = 0.73, p = < 0.001), consistent with the base demographic model (Table 3). Caregivers with care recipients who have not received a diagnosis were less likely to participate (OR = 0.28, *p* = 0.012).

**Table 3:** Estimates from the base model and adjusted demographic model. Characteristics on the left were entered as predictors in a logistic regression model and the outcome measured was hypothetical clinical trial participation

	Base model			Adjusted demographic model		
Characteristics	OR^*1*^	95% CI^*1*^	*p*-value	OR^*1*^	95% CI^*1*^	*p*-value
**Caregiver age (by decade)**	0.72	0.62, 0.84	**< 0.001**	0.73	0.62, 0.87	**< 0.001**
**Caregiver gender: men (compared to women)**	1.32	0.81, 2.17	0.27	1.24	0.74, 2.05	0.41
**Caregiver race/ethnicity**						
White, non-Hispanic	–	–		–	–	
Black, non-Hispanic	0.92	0.53, 1.61	0.77	0.90	0.51, 1.61	0.73
Hispanic	0.49	0.24, 1.01	0.054	0.50	0.24, 1.05	0.066
**Caregiver rurality**	0.64	0.32, 1.29	0.21	0.67	0.33, 1.35	0.26
**Care recipient age (by decade)**				0.99	0.78, 1.28	0.96
**Care recipient gender: men (compared to women)**				0.94	0.56, 1.60	0.83
**Dementia stage**						
Very mild				–	–	
Mild				0.68	0.38, 1.23	0.21
Moderate				0.85	0.39, 1.84	0.68
Advanced				0.84	0.39, 1.82	0.65
**Alzheimer diagnosis**				1.87	0.88, 3.96	0.10
**Dementia diagnosis only**				1.11	0.51, 2.42	0.80
**MCI diagnosis**				0.60	0.21, 1.69	0.33
**No diagnosis**				0.28	0.11, 0.75	**0.012**
**Diagnosis unknown to respondent**				1.45	0.57, 3.70	0.44
**Care recipient rurality**				1.18	0.70, 1.98	0.54

^*1*^ *OR* = Odds Ratio, *CI* = Confidence Interval

### Reasons model

In the reasons model (Table 4), older dementia caregivers were again less likely to participate in the clinical trial (OR = 0.69, p = < 0.001); no significant differences were observed for caregiver gender, race/ethnicity, and rurality. Caregivers who rated social responsibility to participate in research as an important consideration were more likely to participate (OR = 1.56, *p* = 0.049). Caregivers who rated the possibility of the care recipient having serious side effects as important were less likely to participate (OR = 0.51, *p* = 0.003). The rated importance of the possibility of clinical benefit (OR = 1.70, *p* = 0.063) and benefit to one’s own community (OR = 1.66, *p* = 0.070) approached statistical significance as predictors of participation.

**Table 4:** Estimates from the reasons model. Characteristics on the left were entered as predictors in a logistic regression model and the outcome measured was hypothetical clinical trial participation

Reasons Model			
Characteristics	OR^*1*^	95% CI^*1*^	*p*-value
**Caregiver age (by decade)**	0.69	0.58, 0.82	**< 0.001**
**Caregiver gender: men (compared to women)**	1.40	0.80, 2.45	0.23
**Caregiver race/ethnicity**			
White, non-Hispanic	–	–	
Black, non-Hispanic	0.82	0.40, 1.65	0.57
Hispanic	0.48	0.20, 1.15	0.10
**Caregiver rurality**	0.70	0.30, 1.64	0.41
**Trial considerations**			
Clinical benefit	1.70	0.97, 2.96	0.063
Social responsibility to participate in research	1.56	1.00, 2.43	**0.049**
Community benefit	1.66	0.96, 2.88	0.070
Caregiving support	1.38	0.85, 2.24	0.19
Distrust towards the drug company	0.92	0.66, 1.29	0.65
Inconvenience	0.91	0.68, 1.23	0.54
Chance of receiving placebo	0.87	0.64, 1.19	0.40
Risk of side effects	0.51	0.32, 0.80	**0.003**
Privacy concerns	1.14	0.83, 1.56	0.43

The influence of caregiver age on clinical trial participation was not mediated by any of the consideration predictors from the reasons model.

In an exploratory test of effect moderation, there was no significant interaction between the contrast between Hispanic and non-Hispanic caregivers and any of the nine considerations tested. However, interactions between the contrast between non-Hispanic Black and non-Hispanic White caregivers and the rated importance of caregiver support (OR = 0.35, *p* = 0.033) and the possibility of side effects (OR = 2.83, *p* = 0.023) were significant, and between this contrast and benefit to one’s specific community (OR = 3.59, *p* = 0.051) were nearly significant (Supplementary Sect. 2, Additional File 1). In exploratory tests of the reasons model restricted to members of each racial or ethnic subgroup, trial participation among non-Hispanic Black caregivers was predicted by the rated importance of benefit to one’s own community (OR = 5.79, *p* = 0.002) and the possibility of receiving placebo (OR = 0.53, *p* = 0.031); among non-Hispanic White caregivers was predicted by the rated importance of the risk of side effects (OR = 0.34, *p* = 0.002); and was not significantly predicted by the rated importance of any listed consideration among Hispanic caregivers (Supplementary Sect. 3, Additional File 1).

## Discussion

In this preregistered, nationally representative survey, Hispanic and non-Hispanic Black dementia caregivers were not less likely than non-Hispanic White dementia caregivers to participate in a hypothetical dementia clinical trial. In a base and an adjusted demographic model, older caregivers were less likely to participate than younger caregivers. The contrast between Hispanic caregivers and non-Hispanic White caregivers in demographic models was nearly significant, but this principally reflected the associations of Hispanic ethnicity and of trial participation with age; while Hispanic caregivers were similarly likely to participate as non-Hispanic White caregivers overall (29% vs. 34%), they were almost significantly less likely to participate than non-Hispanic White caregivers of similar age. Caregivers who rated social responsibility to participate in research as an important consideration were more likely to participate, while caregivers who rated the possibility of serious side effects as an important consideration were less likely to participate. In exploratory analyses, Black caregivers’ hypothetical participation was sensitive to potential benefits to one’s own community (positively) and the risk of receiving placebo (negative); whereas White caregivers’ hypothetical participation was sensitive to the risk of negative side effects from the active intervention.

Our main findings on race and ethnicity indicate that a lack of diversity in dementia clinical trials is not attributable to a reduced willingness to participate among underrepresented groups. While Hispanic caregivers may be less likely to participate than would be predicted given their younger age, this does not translate into meaningful overall differences in willingness to participate. Particularly regarding Black Americans, our findings from a large nationally representative survey contrast with prior studies (typically in smaller, nonrepresentative samples) identifying distrust of investigators and research procedures to be a significant barrier in enrolling Black Americans in dementia clinical trials [7–9]. While trustworthiness and developing rapport are crucial in research with all communities, our findings suggest that the shameful historic legacy of the Tuskegee syphilis study and other research abuses [21] is not a sufficient explanation for the low enrollment of underrepresented populations in dementia clinical trials.

One potential structural explanation for low Black and Hispanic enrollment in clinical trials is that Black and Hispanic Americans with dementia are diagnosed later in the course of disease, if they receive a diagnosis at all, compared to White Americans [22, 23]. Most current clinical trials of disease-modifying treatments for dementia, such as recent in trials of aducanumab and lecanemab [24, 25], restrict participation to patients in very early stages of disease. Another structural contributor to underrepresentation in clinical trials is the presence of strict exclusion criteria for comorbidities, which are more common in Black and Hispanic older adults [26]. In clinical trials for aducanumab, people with stroke, cardiovascular disease, and chronic kidney disease were excluded [27]. Given worse management for chronic health conditions and related disadvantages in social determinants of health, Black and Hispanic older adults experience significant health disparities, which in turn exclude them from dementia clinical trials, in turn yielding poorer evidence to guide their care, perpetuating further health disparities. Furthermore, the underrepresentation of Black and Hispanic dementia clinical researchers may mean that, even if Black and Hispanic Americans are willing overall to participate in dementia clinical research, the specific recruitment strategies applied in a particular study are less likely to be culturally congruent or to address community-specific barriers to participation (such as lost wages for younger adult caregivers needed as study partners). Finally, participatory methods for community engagement and partnership with community-based organizations are less utilized and less understood among dementia researchers than in many other fields [28]; dementia researchers could improve our work by incorporating lessons from these fields. Particularly among Black caregivers, such strategies may be especially powerful for those who are motivated by the prospect of benefits to their own communities.

Two unanticipated findings from our study warrant further discussion. First, across all demographic groups, only a minority of caregivers (37% overall) would choose to participate in the hypothetical trial. This could represent features of our specific vignette and how the risks and benefits of participation were framed, but disease-modifying therapies currently under investigation are highly burdensome with frequent infusions and complex monitoring requirements, and these features themselves limit participation. Second, in our study, older caregivers are less likely to participate than younger caregivers, contrasting with an earlier study conducted with participants in a single Alzheimer’s Disease Center’s research registry in which older study partners and spouse study partners (highly correlated characteristics) were more likely to participate in a hypothetical dementia clinical trial [29]. While the perspectives of members of research registries are important for immediate success in enrollment for upcoming trials, efforts to expand participation in research will depend more on the perspectives of people not currently included in research efforts. At present, dementia clinical research relies heavily on spouse caregivers, in part because adult child caregivers and other caregivers may face logistical hurdles due to full-time employment or young children for whom they also provide care [30]; given this historic reliance, it is also likely that many studies’ features are structured on the assumption that the study partner is a spouse who lives with the patient. Our findings suggest that addressing logistical and other barriers to participation by younger caregivers could enhance study enrollment overall. It is also noteworthy that in our nationally representative sample only a very small minority of dementia caregivers (6%) are spouse caregivers, which is comparable to previous nationally representative samples [31].

Our study has several limitations. While we used representative sampling techniques with deliberate oversampling for Hispanic and non-Hispanic Black caregivers, we did not have the ability to oversample other groups that are underrepresented in dementia research, such as Asian Americans [32, 33], and groups that are underrepresented and at increased risk for dementia, such as Indigenous Americans [7, 34, 35]. More focused work must be done to address specific barriers to research participation in these communities. Additionally, while we used an existing population-based survey platform to facilitate the demographic representativeness of our sample, and while the NORC AmeriSpeak panel uses a multi-stage recruitment process to limit nonresponse bias, people who agree to join a panel and then respond to surveys on that panel may differ from those who do not. As our study specifically addresses willingness to participate in research, some motivations for nonparticipation may not be reflected using these techniques (or potentially, any techniques that depend on informed consent). Our use of a relatively large, population-based sample enhanced our ability to detect and generalize across group differences, but lacked the flexibility of qualitative or mixed-methods approaches to explore individual participants’ reasons for participation or nonparticipation.

## Conclusion

In summary, our results indicate that low enrollment of underrepresented populations in dementia clinical trials is not attributable to less willingness among dementia caregivers from underrepresented groups; such under enrollment instead likely reflects structural barriers and historic exclusion from trial participation. The lack of diversity in dementia clinical trials may not only undermine generalizability but also exacerbate health disparities. These findings emphasize the importance of improving recruitment strategies and conducting more focused work to addressing structural barriers that hinder non-Hispanic Black and Hispanic populations from participating. It is imperative that researchers reach out to these populations, before symptoms present and in the early stages of the disease, through continuous community outreach and engagement, while also being receptive to their needs to help mitigate the health disparities underrepresentation has contributed to.

## Electronic supplementary material

Below is the link to the electronic supplementary material.

Supplementary Material 1

## Data Availability

The datasets generated and analyzed during the current study are available in the Open Science Framework repository at https://osf.io/je7fs/.
