# Supplementary material for: Perceptions about dementia clinical trials among underrepresented populations: a nationally representative survey of U.S. dementia caregivers
Source: Alzheimers Res Ther. 2024 Oct 15;16:224. doi: 10.1186/s13195-024-01579-5 (PMC11476697; doi:10.1186/s13195-024-01579-5)
Supplement: Supplementary file 1 — Supplementary Material 1 [file 13195_2024_1579_MOESM1_ESM.docx]

**Caregiver survey supplementary materials additional file 1**

Manuscript Title: Perceptions about dementia clinical trials among underrepresented populations: A nationally representative survey of U.S. dementia caregivers

Manuscript Authors: Brandon Leggins, Danielle M. Hart, Ashley J. Jackson, Robert W. Levenson, Charles C. Windon, Jennifer Merrilees, Winston Chiong.

**Supplementary section 1**

Hypothetical dementia clinical trial vignette text:

*Key:*

*[Q9_INSERT] = the person the caregiver indicated they [care/cared] for (i.e., grandparent, parent, sibling, spouse, another relative, friend, or neighbor)*

*[DOV_Q14TOP] = the care recipient’s dementia diagnosis*

*Past tense wording (as opposed to present tense) was provided for caregivers who had provided care in the preceding 12 months but were not currently providing care at the time of the survey*

Imagine that you [take/had taken] your [Q9_INSERT] to their doctor's appointment and the doctor [tells/had told] both of you about a new research study to test an experimental medication called Cognemab to treat [DOV_Q14TOP].

The researchers [want/wanted] to find out whether taking Cognemab will slow down the progression of [DOV_Q14TOP]. They [do/did] not think Cognemab [will/would] make your [Q9_INSERT]'s memory or thinking better, or keep it the same as it [is now/was]. Instead, the study [will show/would have shown] whether people's memory and thinking get worse more slowly with Cognemab than without it.

Half of the study participants [will/would] receive injections of Cognemab and half [will/would] receive injections of a placebo (an inactive substance). If your [Q9_INSERT] [enrolls/had enrolled] in the study, they [will have/would have had] a 50/50 chance of being put in either group, and neither they nor their own doctor [will find/would have found] out which group they [are/were] in until the end of the study.

The study [will last/would have lasted] 18 months. If your [Q9_INSERT] [enrolls/had enrolled] in the study, both of you [will go/would have gone] to a research center in the nearest major city together every month. Your [Q9_INSERT] [will get/would have gotten] monthly injections of either Cognemab or placebo, and every six months [will take/would have taken] memory tests, [get/gotten] blood tests, and [have/had] a brain scan. You [will answer/would have answered] questions about how your [Q9_INSERT] [is/was] doing every month. You [will get/would have gotten] $20 per visit and the company [will pay/would have paid] for travel expenses like gas, bus/train fare, and parking.

As possible benefits to being in the study, there [is/was] a 50% chance that your [Q9_INSERT] [will receive/would have received] Cognemab. The researchers [do/did] not know if Cognemab works, but if Cognemab does work, your [Q9_INSERT] could [live/have lived] and [keep/kept] their memory for several months longer if they [get/received] Cognemab than they would otherwise. Even if Cognemab does not work, participating [will help/would have helped] researchers to learn more to help other people with [DOV_Q14TOP] in the future.

As possible risks, there [is/was] a 50% chance that your [Q9_INSERT] [will receive/would have received] the placebo, in which case you [will both/would both have] come for monthly research visits with no expected benefit to your [Q9_INSERT]. Cognemab also has risks. 1 in 3 patients who get Cognemab develop brain swelling, which the researchers [will/would] monitor with brain scans for safety. Usually this brain swelling does not cause noticeable problems, but 1 in 10 patients who get Cognemab have side effects like headache, nausea or confusion and need to stop taking Cognemab for a while. 1 in 100 patients who get Cognemab have serious side effects requiring special treatment, sometimes in the hospital. The company that makes Cognemab [will pay/would have paid] any medical expenses.

In summary:

- There is a new study testing if Cognemab, an experimental medication, will slow down the progression of [DOV_Q14TOP].
- Half of the study participants [will/would] receive Cognemab and half [will/would] receive a placebo (an inactive substance).
- Possible benefits: 50% chance that your [Q9_INSERT] [will receive/would have received] Cognemab. If Cognemab does work, your [Q9_INSERT] could [live/have lived] and [keep/kept] their memory for several months longer.
- Possible risks: 50% chance that your [Q9_INSERT] [will receive/would have received] the placebo. Cognemab also has risks. 1 in 3 patients who get Cognemab develop brain swelling, which the researchers [will/would] monitor with brain scans for safety.

Vignette response options:

Based on this information, would you [participate/have participated] in this study with your [Q9_INSERT]?

1. Definitely yes
2. Probably yes
3. Probably no
4. Definitely no

**Supplementary section 2: Test of moderation that includes interaction effects between trial considerations (reasons model predictors) and race/ethnicity**

| **Interaction of Race/Ethnicity and Reasons** | | | |
| --- | --- | --- | --- |
| **Characteristic** | **OR***^1^* | **95% CI***^1^* | **p-value** |
| **Caregiver age** | 0.67 | 0.55, 0.80 | **<0.001** |
| **Caregiver gender: men (compared to women)** | 1.63 | 0.92, 2.88 | 0.094 |
| **Caregiver race/ethnicity** |  |  |  |
| White, non-Hispanic | — | — |  |
| Black, non-Hispanic | 0.79 | 0.34, 1.88 | 0.60 |
| Hispanic | 0.37 | 0.07, 1.90 | 0.23 |
| **Trial considerations** |  |  |  |
| Clinical benefit | 2.02 | 0.93, 4.39 | 0.076 |
| Social responsibility to participate in research | 2.17 | 1.02, 4.60 | **0.044** |
| Community benefit | 1.33 | 0.58, 3.07 | 0.50 |
| Caregiving support | 1.63 | 0.86, 3.09 | 0.14 |
| Distrust towards the drug company | 1.02 | 0.64, 1.62 | 0.94 |
| Inconvenience | 0.91 | 0.61, 1.37 | 0.66 |
| Chance of receiving placebo | 0.82 | 0.52, 1.28 | 0.38 |
| Risk of side effects | 0.35 | 0.18, 0.67 | **0.002** |
| Privacy concerns | 1.29 | 0.82, 2.02 | 0.27 |
| **Caregiver race/ethnicity * Clinical benefit** |  |  |  |
| Black, non-Hispanic * Clinical benefit | 0.39 | 0.14, 1.09 | 0.071 |
| Hispanic * Clinical benefit | 1.26 | 0.27, 5.90 | 0.77 |
| **Caregiver race/ethnicity * Social responsibility** |  |  |  |
| Black, non-Hispanic * Social responsibility | 0.63 | 0.25, 1.64 | 0.35 |
| Hispanic * Social responsibility | 0.55 | 0.16, 1.82 | 0.32 |
| **Caregiver race/ethnicity * Community benefit** |  |  |  |
| Black, non-Hispanic * Community benefit | 3.59 | 0.99, 13.0 | 0.051 |
| Hispanic * Community benefit | 1.02 | 0.24, 4.31 | 0.98 |
| **Caregiver race/ethnicity * Caregiving support** |  |  |  |
| Black, non-Hispanic * Caregiving support | 0.35 | 0.13, 0.92 | **0.033** |
| Hispanic * Caregiving support | 0.73 | 0.15, 3.61 | 0.70 |
| **Caregiver race/ethnicity * Distrust** |  |  |  |
| Black, non-Hispanic * Distrust | 1.28 | 0.63, 2.59 | 0.50 |
| Hispanic * Distrust | 0.82 | 0.32, 2.14 | 0.69 |
| **Caregiver race/ethnicity * Inconvenience** |  |  |  |
| Black, non-Hispanic * Inconvenience | 0.70 | 0.37, 1.33 | 0.28 |
| Hispanic * Inconvenience | 1.09 | 0.51, 2.35 | 0.83 |
| **Caregiver race/ethnicity * Placebo** |  |  |  |
| Black, non-Hispanic * Placebo | 0.70 | 0.34, 1.43 | 0.33 |
| Hispanic * Placebo | 1.79 | 0.71, 4.51 | 0.22 |
| **Caregiver race/ethnicity * Side effect** |  |  |  |
| Black, non-Hispanic * Side effect | 2.83 | 1.16, 6.93 | **0.023** |
| Hispanic * Side effect | 1.71 | 0.45, 6.53 | 0.43 |
| **Caregiver race/ethnicity * Privacy** |  |  |  |
| Black, non-Hispanic * Privacy | 0.96 | 0.47, 1.98 | 0.91 |
| Hispanic * Privacy | 0.51 | 0.21, 1.24 | 0.14 |
| *^1^* OR = Odds Ratio, CI = Confidence Interval | | | |

**Supplementary section 3:** **exploratory tests of the reasons model restricted to members of each racial or ethnic subgroup.**

| **Reasons Model in non-Hispanic Black Caregivers** | | | | |
| --- | --- | --- | --- | --- |
| **Characteristic** | **OR***^1^* | **95% CI***^1^* | **p-value** | **VIF***^1^* |
| **Caregiver age** | 0.55 | 0.39, 0.77 | **<0.001** | 1.2 |
| **Caregiver gender: men (compared to women)** | 2.44 | 0.83, 7.18 | 0.11 |  |
| **Caregiver rurality** |  |  |  | 1.2 |
| Urban/Suburban | — | — |  |  |
| Rural | 0.87 | 0.16, 4.76 | 0.87 |  |
| **Clinical benefit** | 0.77 | 0.39, 1.51 | 0.44 | 2.1 |
| **Social responsibility** | 1.40 | 0.77, 2.54 | 0.27 | 1.7 |
| **Community benefit** | 5.79 | 1.96, 17.1 | **0.002** | 4.0 |
| **Caregiving support** | 0.50 | 0.23, 1.08 | 0.079 | 2.8 |
| **Distrust** | 1.35 | 0.77, 2.37 | 0.30 | 1.7 |
| **Inconvenience** | 0.62 | 0.36, 1.09 | 0.10 | 1.7 |
| **Placebo** | 0.53 | 0.29, 0.94 | **0.031** | 1.8 |
| **Side effect** | 1.02 | 0.54, 1.93 | 0.94 | 2.0 |
| **Privacy** | 1.22 | 0.68, 2.18 | 0.50 | 1.8 |
| *^1^* OR = Odds Ratio, CI = Confidence Interval, VIF = Variance Inflation Factor | | | | |

| **Reasons Model in non-Hispanic White Caregivers** | | | | |
| --- | --- | --- | --- | --- |
| **Characteristic** | **OR***^1^* | **95% CI***^1^* | **p-value** | **VIF***^1^* |
| **Caregiver age** | 0.63 | 0.50, 0.79 | **<0.001** | 1.3 |
| **Caregiver gender: men (compared to women)** | 2.00 | 0.91, 4.36 | 0.083 |  |
| **Caregiver rurality** |  |  |  | 1.3 |
| Urban/Suburban | — | — |  |  |
| Rural | 0.62 | 0.23, 1.67 | 0.34 |  |
| **Clinical benefit** | 1.95 | 0.89, 4.24 | 0.094 | 3.2 |
| **Social responsibility** | 2.20 | 0.97, 4.96 | 0.058 | 2.4 |
| **Community benefit** | 1.35 | 0.58, 3.14 | 0.49 | 3.1 |
| **Caregiving support** | 1.64 | 0.84, 3.20 | 0.15 | 3.2 |
| **Distrust** | 1.03 | 0.64, 1.65 | 0.90 | 1.9 |
| **Inconvenience** | 0.91 | 0.59, 1.40 | 0.67 | 1.4 |
| **Placebo** | 0.84 | 0.53, 1.32 | 0.45 | 2.0 |
| **Side effect** | 0.34 | 0.17, 0.66 | **0.002** | 2.5 |
| **Privacy** | 1.31 | 0.83, 2.08 | 0.25 | 1.9 |
| *^1^* OR = Odds Ratio, CI = Confidence Interval, VIF = Variance Inflation Factor | | | | |

| **Reasons Model in Hispanic Caregivers** | | | | |
| --- | --- | --- | --- | --- |
| **Characteristic** | **OR***^1^* | **95% CI***^1^* | **p-value** | **VIF***^1^* |
| **Caregiver age** | 0.88 | 0.53, 1.47 | 0.63 | 2.0 |
| **Caregiver gender: men (compared to women)** | 0.63 | 0.17, 2.32 | 0.48 |  |
| **Caregiver rurality** |  |  |  | 1.7 |
| Urban/Suburban | — | — |  |  |
| Rural | 1.29 | 0.18, 9.18 | 0.79 |  |
| **Clinical benefit** | 2.13 | 0.66, 6.84 | 0.20 | 3.1 |
| **Trial responsibility** | 1.25 | 0.58, 2.69 | 0.56 | 1.9 |
| **Community benefit** | 1.15 | 0.47, 2.83 | 0.76 | 1.8 |
| **Caregiving support** | 1.41 | 0.43, 4.65 | 0.57 | 3.3 |
| **Distrust** | 0.86 | 0.38, 1.93 | 0.71 | 1.8 |
| **Inconvenience** | 0.90 | 0.52, 1.58 | 0.72 | 1.5 |
| **Placebo** | 1.35 | 0.67, 2.73 | 0.40 | 1.4 |
| **Side effect** | 0.55 | 0.18, 1.69 | 0.29 | 1.8 |
| **Privacy** | 0.76 | 0.38, 1.52 | 0.44 | 1.9 |
| *^1^* OR = Odds Ratio, CI = Confidence Interval, VIF = Variance Inflation Factor | | | | |

**Supplementary section 4: Outputs of model 3 and model 4.**

| **Model 3** | | | | |
| --- | --- | --- | --- | --- |
| **Characteristic** | **OR***^1^* | **95% CI***^1^* | **p-value** | **GVIF***^1^* |
| **Caregiver age** | 0.64 | 0.52, 0.79 | **<0.001** | 2.1 |
| **Caregiver gender: men (compared to women)** | 1.18 | 0.69, 2.00 | 0.54 | 1.2 |
| **Caregiver race/ethnicity** |  |  |  | 1.7 |
| White, non-Hispanic | — | — |  |  |
| Black, non-Hispanic | 1.08 | 0.58, 2.01 | 0.80 |  |
| Hispanic | 0.45 | 0.21, 1.00 | **0.050** |  |
| **Caregiver rurality** |  |  |  | 1.4 |
| Urban/Suburban | — | — |  |  |
| Rural | 0.60 | 0.28, 1.28 | 0.18 |  |
| **Care recipient age** | 1.03 | 0.79, 1.33 | 0.85 | 1.6 |
| **Care recipient gender: men (compared to women)** | 0.99 | 0.57, 1.73 | 0.97 | 1.3 |
| **Dementia Stage** |  |  |  | 1.6 |
| Very mild | — | — |  |  |
| Mild | 0.81 | 0.43, 1.53 | 0.52 |  |
| Moderate | 0.92 | 0.41, 2.08 | 0.85 |  |
| Advanced | 0.90 | 0.41, 1.98 | 0.79 |  |
| **Alzheimer diagnosis** |  |  |  | 2.3 |
| No | — | — |  |  |
| Yes | 1.99 | 0.86, 4.59 | 0.11 |  |
| **Dementia only diagnosis** |  |  |  | 2.2 |
| No | — | — |  |  |
| Yes | 1.28 | 0.55, 2.96 | 0.57 |  |
| **MCI diagnosis** |  |  |  | 1.7 |
| No | — | — |  |  |
| Yes | 0.66 | 0.22, 1.96 | 0.46 |  |
| **No diagnosis from doctor** |  |  |  | 2.0 |
| No | — | — |  |  |
| Yes | 0.31 | 0.11, 0.93 | **0.036** |  |
| **Care recipient received diagnosis from doctors but caregiver doesn’t know** |  |  |  | 1.6 |
| No | — | — |  |  |
| Yes | 1.08 | 0.40, 2.87 | 0.88 |  |
| **Caregiver education** | 1.07 | 0.82, 1.40 | 0.63 | 1.7 |
| **Caregiver income (scaled by $10k)** | 0.99 | 0.93, 1.04 | 0.61 | 1.5 |
| **Caregiver employment** |  |  |  | 2.5 |
| Working | — | — |  |  |
| Retired | 1.30 | 0.51, 3.33 | 0.58 |  |
| Disabled | 0.81 | 0.30, 2.14 | 0.67 |  |
| Other unemployed | 0.68 | 0.33, 1.38 | 0.28 |  |
| **Care recipient rurality** |  |  |  | 1.4 |
| Urban/Suburban | — | — |  |  |
| Rural | 1.23 | 0.71, 2.14 | 0.47 |  |

| **Model 4** | | | | |
| --- | --- | --- | --- | --- |
| **Characteristic** | **OR***^1^* | **95% CI***^1^* | **p-value** | **GVIF***^1^* |
| **Caregiver age** | 0.60 | 0.47, 0.75 | **<0.001** | 2.7 |
| **Caregiver gender: men (compared to women)** | 1.28 | 0.75, 2.18 | 0.36 | 1.3 |
| **Caregiver race/ethnicity** |  |  |  | 1.9 |
| White, non-Hispanic | — | — |  |  |
| Black, non-Hispanic | 1.14 | 0.60, 2.15 | 0.68 |  |
| Hispanic | 0.48 | 0.22, 1.06 | 0.069 |  |
| **Caregiver rurality** |  |  |  | 1.5 |
| Urban/Suburban | — | — |  |  |
| Rural | 0.59 | 0.27, 1.27 | 0.17 |  |
| **Care recipient age** | 1.12 | 0.86, 1.46 | 0.39 | 1.7 |
| **Care recipient gender: men (compared to women)** | 0.97 | 0.53, 1.75 | 0.91 | 1.5 |
| **Dementia stage** |  |  |  | 1.8 |
| Very mild | — | — |  |  |
| Mild | 0.88 | 0.47, 1.66 | 0.69 |  |
| Moderate | 1.16 | 0.51, 2.67 | 0.72 |  |
| Advanced | 1.05 | 0.47, 2.36 | 0.91 |  |
| **Alzheimer diagnosis** |  |  |  | 2.4 |
| No | — | — |  |  |
| Yes | 1.79 | 0.77, 4.13 | 0.17 |  |
| **Dementia only diagnosis** |  |  |  | 2.3 |
| No | — | — |  |  |
| Yes | 1.26 | 0.53, 2.98 | 0.60 |  |
| **MCI diagnosis** |  |  |  | 1.8 |
| No | — | — |  |  |
| Yes | 0.56 | 0.18, 1.69 | 0.30 |  |
| **No diagnosis from doctor** |  |  |  | 2.1 |
| No | — | — |  |  |
| Yes | 0.33 | 0.11, 1.02 | 0.054 |  |
| **Care recipient received diagnosis from doctors, but caregiver doesn’t know** |  |  |  | 1.8 |
| No | — | — |  |  |
| Yes | 0.93 | 0.36, 2.39 | 0.89 |  |
| **Caregiver education** | 1.13 | 0.85, 1.49 | 0.41 | 1.9 |
| **Caregiver income (scaled by $10k)** | 0.99 | 0.93, 1.05 | 0.63 | 1.6 |
| **Caregiver employment** |  |  |  | 2.9 |
| Working | — | — |  |  |
| Retired | 1.51 | 0.60, 3.79 | 0.38 |  |
| Disabled | 0.88 | 0.32, 2.44 | 0.81 |  |
| Other unemployed | 0.69 | 0.33, 1.44 | 0.33 |  |
| **Relationship (who is the person you cared for)** |  |  |  | 2.8 |
| Other relative | — | — |  |  |
| Parent | 1.79 | 0.95, 3.37 | 0.074 |  |
| Spouse/partner | 1.01 | 0.20, 5.13 | >0.99 |  |
| Non-relative | 1.64 | 0.78, 3.45 | 0.19 |  |
| **Care recipient residence** |  |  |  | 3.3 |
| In home with caregiver | — | — |  |  |
| In a separate home | 0.63 | 0.27, 1.48 | 0.29 |  |
| Long term/residential | 0.55 | 0.18, 1.66 | 0.29 |  |
| **Primary caregiver** |  |  |  | 1.5 |
| No | — | — |  |  |
| Yes | 1.77 | 0.94, 3.34 | 0.079 |  |
| **Visit frequency** | 0.90 | 0.68, 1.19 | 0.45 | 2.3 |
| **Care recipient rurality** |  |  |  | 1.6 |
| Urban/Suburban | — | — |  |  |
| Rural | 1.21 | 0.66, 2.21 | 0.53 |  |
